# Spinal Cord Compression as Initial Presentation in Hepatocellular Carcinoma

**DOI:** 10.7759/cureus.16238

**Published:** 2021-07-07

**Authors:** Jasmeet Kaur, Ambreen Malik, Mehrvaan Kaur, Judie Goodman

**Affiliations:** 1 Internal Medicine, Saint Joseph Mercy Oakland Hospital, Pontiac, USA; 2 Diagnostic Radiology, Saint Joseph Mercy Oakland Hospital, Pontiac, USA; 3 Hematology and Medical Oncology, Saint Joseph Mercy Oakland Hospital, Pontiac, USA

**Keywords:** hepatocellular carcinoma (hcc), unresectable hcc, spinal cord compression, radiotherapy (rt), kyphoplasty

## Abstract

Hepatocellular carcinoma (HCC) is the most common primary hepatic malignancy and aggressive neoplasms with high metastatic potential to the lung, regional lymph nodes, and bone. Metastatic spinal cord compression due to HCC is a rare, unusual initial presentation and is a neurosurgical emergency. We present two cases of HCC where spinal cord compression was the initial presentation before the diagnosis of HCC. Our first patient presented with bilateral flank pain. The biopsy of the chest wall showed HCC. However, a CT scan of the abdomen showed metastatic involvement of the T11 vertebra. He was found to have severe spinal canal stenosis on MRI of the back without neurological deficit. He underwent an emergent tumor decompression of the T11 vertebra with kyphoplasty. Our second patient was a young man who presented with cord compression symptoms, which included bilateral leg weakness and an inability to void. An urgent laminectomy with decompression of the lumbar spine was performed. He received radiotherapy for spinal metastasis and started systemic therapy for HCC. Unfortunately, he failed multiple lines of systemic therapy, and the progression of the disease complicated his clinical course with spinal cord compression for the second time.

## Introduction

Hepatocellular carcinoma (HCC) is the most common primary hepatic malignancy and the sixth most common cancer worldwide [1]. It is one of the aggressive neoplasms with a high metastatic potential [2]. The most common metastasis site is the lung (37%-70%), followed by regional lymph nodes (23%-45%) and bones [3]. The most frequent sites of bone metastasis are the ribs, spine, femur, pelvis, and humerus. Metastatic spinal cord compression due to HCC is a rare and unusual initial presentation and is a neurosurgical emergency. We present two cases of HCC where spinal cord compression was the initial presentation even before the diagnosis of HCC and its management.

## Case presentation

Case 1

**Figure 1:**
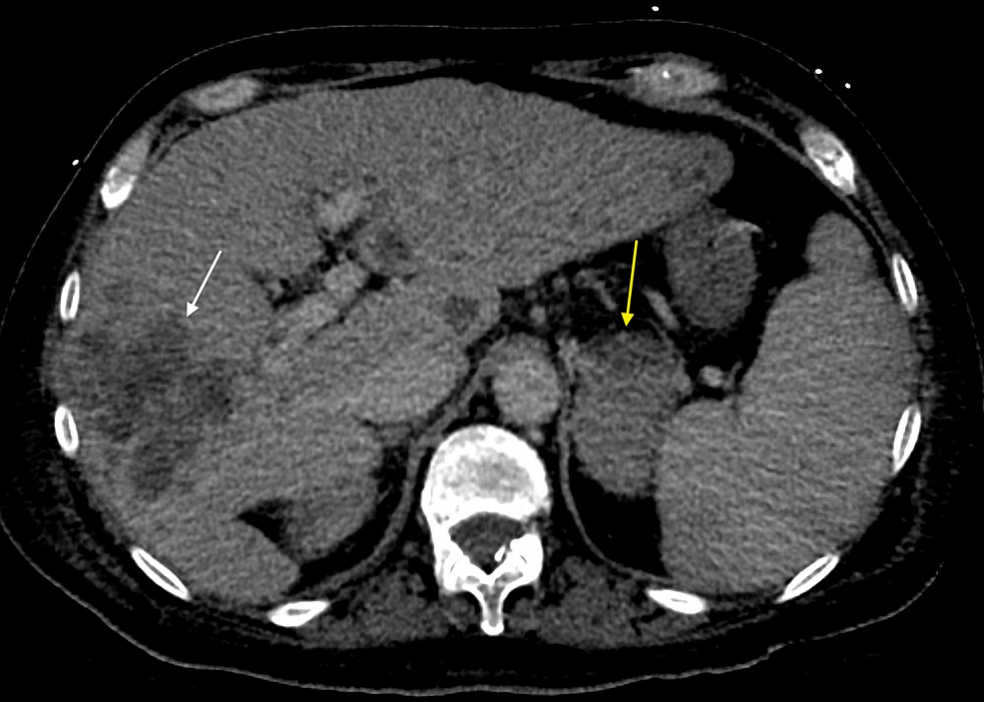
CT abdomen Axial planes of a contrast-enhanced CT abdomen and pelvis in the plane show conglomerate of ill-defined low attenuating lesions in the right hepatic lobe (marked by white arrow) and enlarged left adrenal gland( marked by yellow arrow) consistent with metastasis.

**Figure 2:**
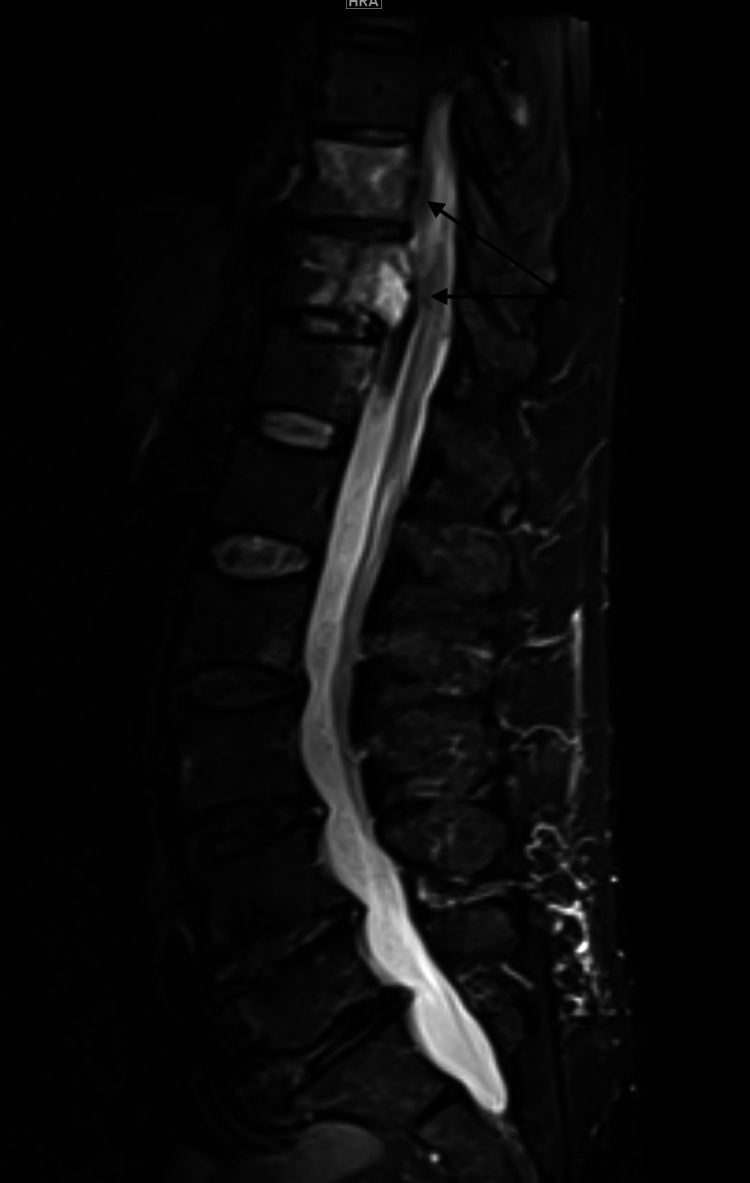
MRI thoracic and lumbar spine Sagittal STIR (Short Tau Inversion Recovery) shows high signal intensity at the T10 and T11 vertebral bodies (marked by the black arrows), with moderate-severe compression of the spinal cord at T11.

**Figure 3:**
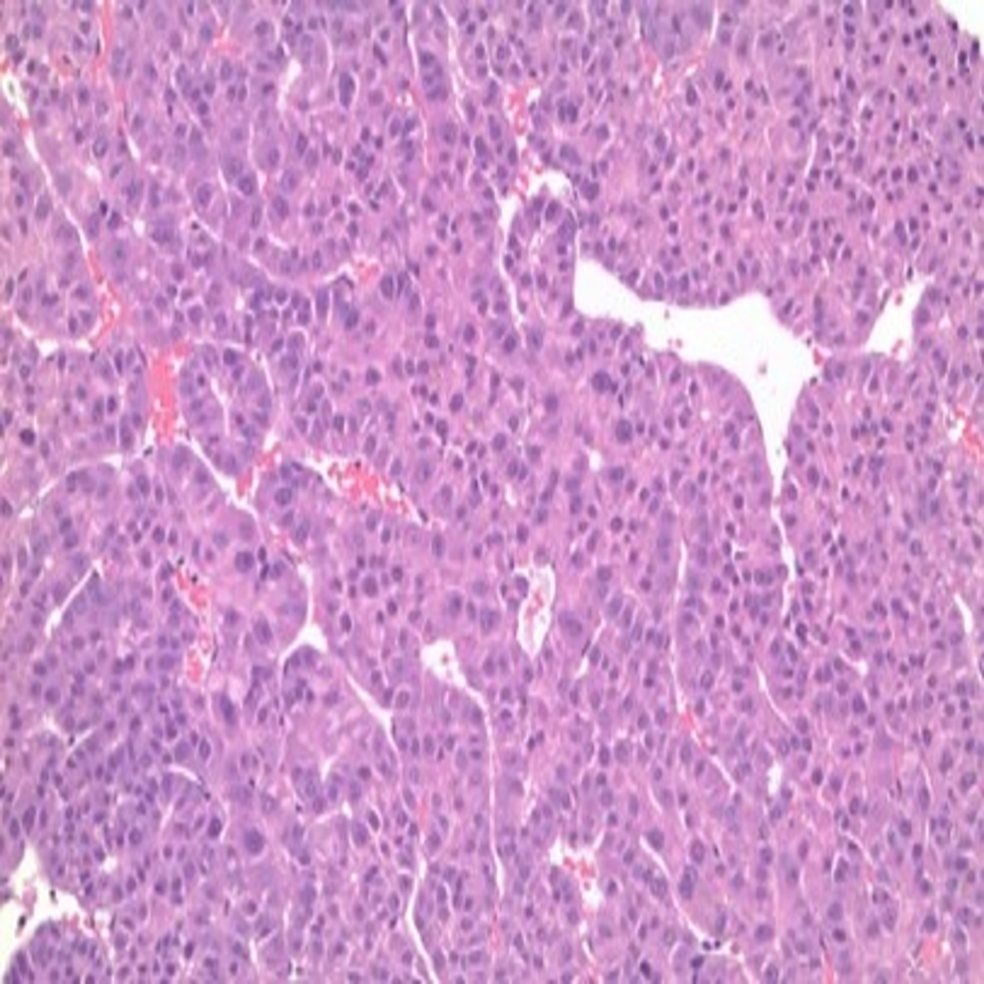
Biopsy chest wall mass Core biopsies of chest wall mass under 40x magnification show a malignant epithelial neoplasm with hepatoid features. Immunohistochemical staining is positive for HSA (hepatocyte-specific antigen), supporting the diagnosis of a moderately differentiated hepatocellular carcinoma.

**Figure 4:**
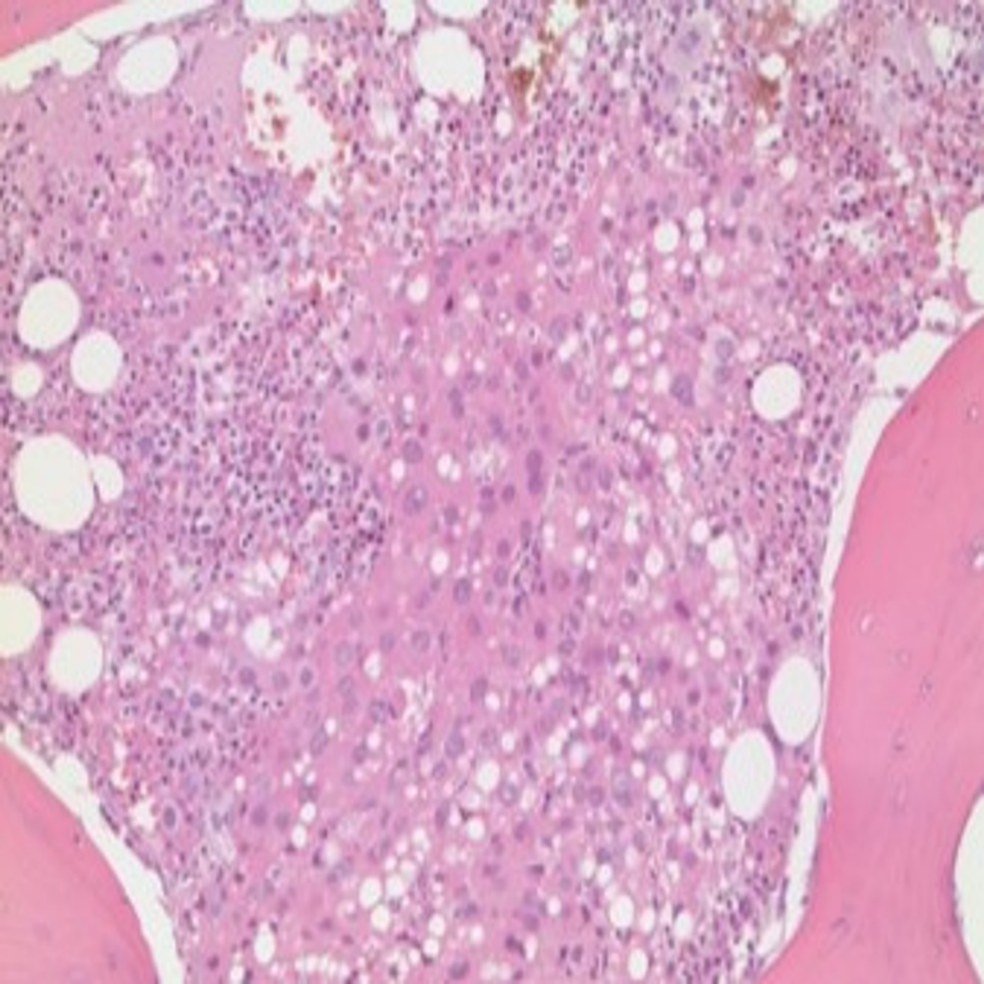
Biopsy vertebrae Histological illustration of lumbar vertebra under 40x magnification showing portions of bone containing foci of metastatic tumor. Immunohistochemical staining was strongly positive for arginase and HSA and focally positive for AFP. These results support the diagnosis of metastatic hepatocellular carcinoma.

A 60-year-old male with a history of heavy alcohol drinking, tobacco smoking, and illicit drug abuse presented with bilateral flank and back pain. On examination, he was hemodynamically stable. The cardiovascular and respiratory system was unremarkable. He had an anterior chest wall mass and spinal tenderness in the lower thoracic region but without neurological deficit on physical examination. Laboratory work-up was positive for hepatitis C serology with a high viral load. CT abdomen and pelvis imaging showed diffuse metastatic disease of the liver, right lung, adrenal gland, and thoracic spine (Figure 1). MRI spine showed retropulsion of the T11 vertebra, causing severe spinal stenosis (Figure 2). Biopsy of the chest wall mass showed moderately differentiated hepatocellular carcinoma (Figure 3). The patient was started on steroids, and he underwent kyphoplasty of the T11 vertebra (biopsy vertebrae shown in Figure 4). The patient received palliative radiation therapy and was treated for hepatitis C. The patient was started on combination therapy with atezolizumab (immune checkpoint inhibitor) and bevacizumab (vascular endothelial growth factor receptor inhibitor), with a desirable response.

Case 2

**Figure 5:**
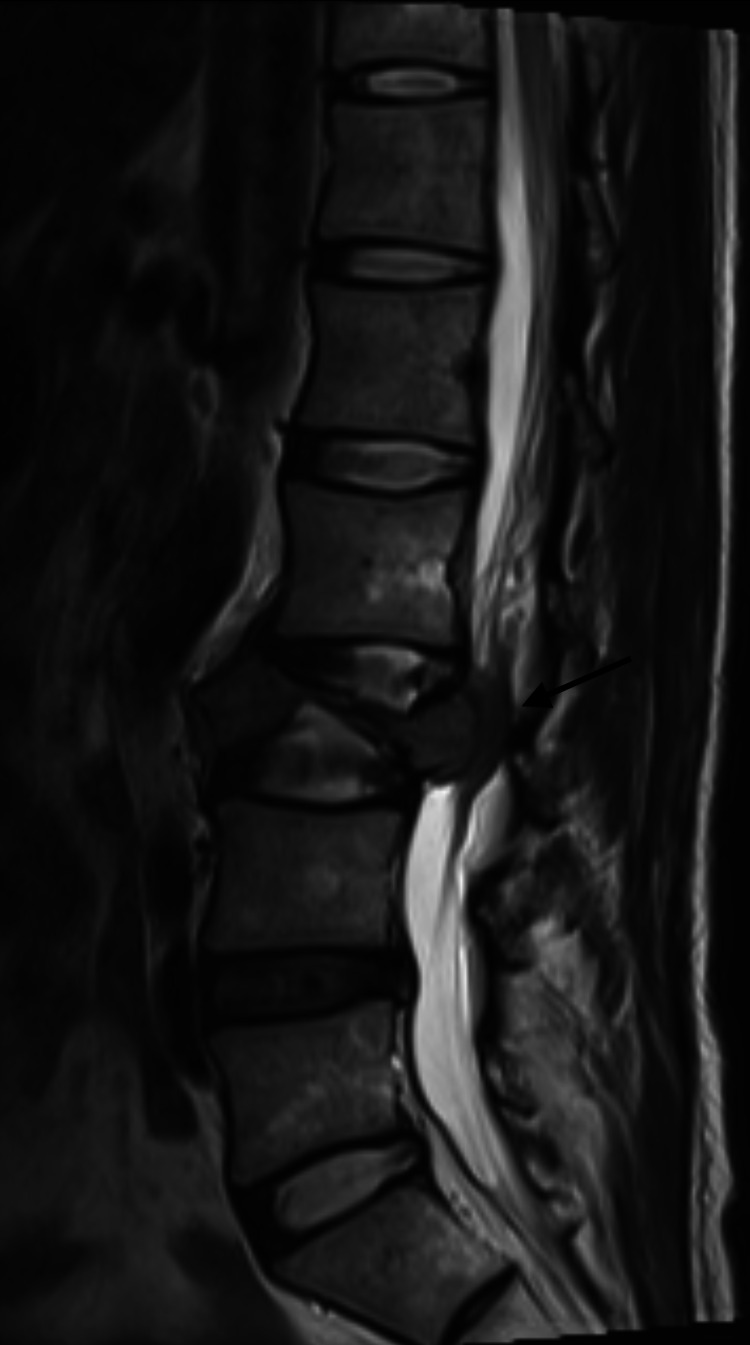
T2 weighted MR lumbar spine Sagittal view of MR lumbar spine at initial presentation. T2 weighted image shows extensive edema signal at the L3 vertebral body and severe retropulsion of the vertebral body causing severe spinal canal stenosis (marked by the black arrow).

**Figure 6:**
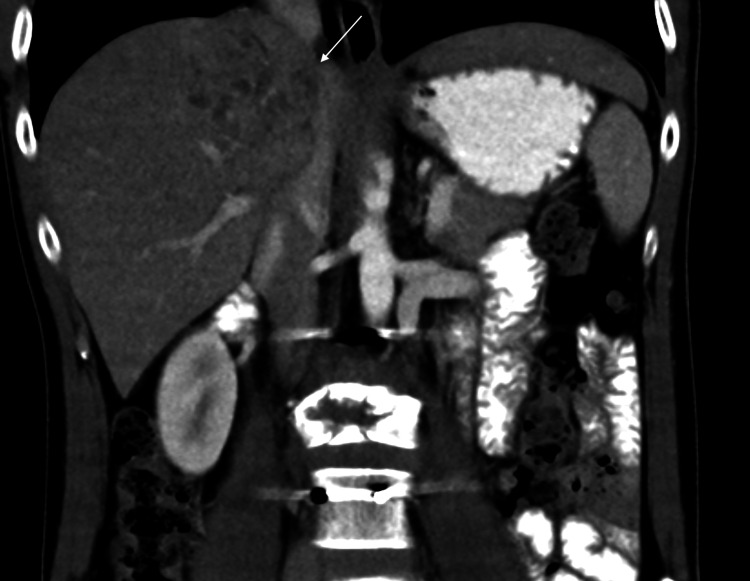
CT abdomen A contrast-enhanced CT scan in the coronal plane shows an ill-defined heterogeneously enhancing right hepatic lobe mass measuring 5 cm in the maximum dimension, which has a mild mass effect on the inferior vena cava (marked by white arrow).

**Figure 7:**
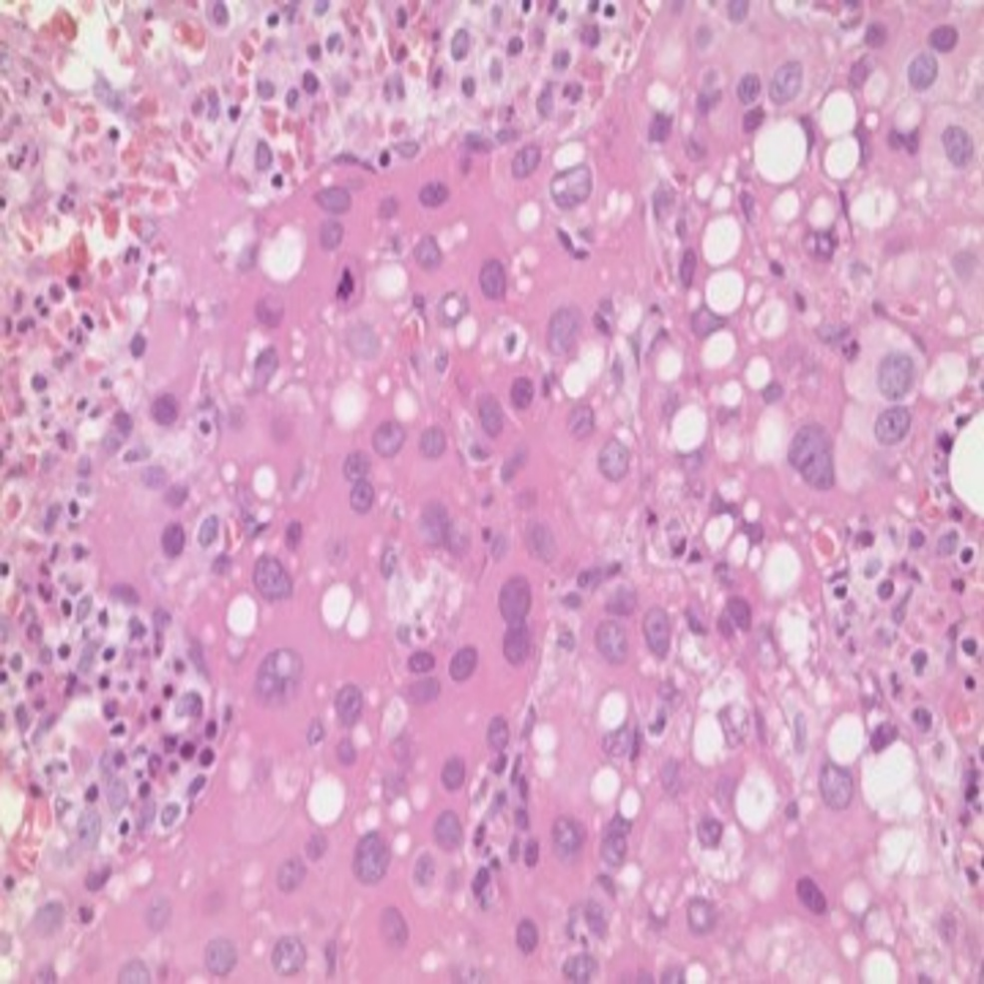
Vertebrae bone biopsy Histological illustration of bone under 40x magnification showing portions of bone containing foci of metastatic tumor.

A 38-year-old Chinese man without a significant past medical or surgical history initially presented with low back pain and leg pain. He reported an inability to void and weakness in his legs. He denies weight loss, abdominal pain, nausea, or vomiting. He did not have a history of smoking, alcohol, or blood transfusion. On physical examination, spinal tenderness was present with reduced strength in both lower extremities. He was Child-Pugh class B. Hepatitis B surface antigen (HBsAg) was positive, but the HCV serology was negative. The AFP was 43519 ng/ml. The MRI of the spine demonstrated a compression fracture of L3 with retropulsion fragment, causing severe spinal canal stenosis (Figure 5). CT scan of the chest, abdomen, and pelvis revealed a mass in the right hepatic lobe measuring 5 cm with mild to moderate mass effect on the intrahepatic inferior vena cava (Figure 6). The patient underwent a bilateral laminectomy of L3, partial laminectomy of L2, and subsequently decompression of the lumbar spine. The biopsy of the bone lesion showed metastatic HCC (Figure 7). He was started on radiotherapy and systemic treatment. He failed multiple systemic agents, including sorafenib, nivolumab, and regorafenib. His disease continued to progress. Two years later, the patient presented again with sudden onset of severe lower extremity weakness with difficulty in urination and bowel movement. Imaging showed severe spinal stenosis with enhancing lesions in the thoracic spine. He was again started on steroids and radiation treatment. CT abdomen and pelvis showed widespread metastasis disease with the lung, liver, and bone lesions. The patient had a progression of the disease, he was not eligible for any further systemic treatment for HCC, and he was in hospice care. The patient expired in the next few days in hospice care.

## Discussion

The improvement in the overall survival and progression-free survival seen in HCC patients with the development of novel systemic therapies and improvement in diagnostic modalities [2]. An increasing number of metastatic HCC are being reported with advancements in diagnostic modalities and prolonged patient survival [2]. In a national database analysis study by Ali Abbas, the prevalence of HCC metastasis is 18%. The lung is the most common site, followed by regional lymph node/peritoneum and bone [2]. The reported incidence of bone metastasis as an initial presentation is 3.3%-5.1% [4]. The most common site for bone metastasis is the spine, followed by the pelvis, ribs, and skull, with the thoracic spine being the most involved [4]. A systematic review by C Rory Goodwin with a total of 152 patients diagnosed with HCC with spinal metastasis showed the median time to the spine metastasis from the time of diagnosis of the primary tumor was 20 months (±10.21 months) [3]. Bone metastasis rarely can manifest symptoms even before clinical manifestations of liver disease. Spinal cord compression as the initial presentation of HCC is exceedingly rare. In a case report by Liaukovich, two patients of age 64 years and 70 years, male, initially presented with the symptoms of spinal cord compression due to metastasis of HCC [5]. In a review of a series of 600 patients with spinal cord compression, HCC has been reported in less than 2% of cases, making it an infrequent feature of HCC [6]. Here we report two cases of HCC with spinal cord compression as the initial presentation before HCC was diagnosed and treatment course. Back pain is the most common presenting symptom of bone metastasis due to the regional tumor involvement of the nerve root. The rapid tumor growth, surrounding edema, or tumor hemorrhage can compress the spinal vascular supply and spinal cord with worsening neurologic signs and symptoms [7]. Early detection of neurologic symptoms and treatment is necessary to prevent worsening neurologic function and complete paralysis [3,8]. Glucocorticoid is part of the standard regimen for symptomatic spinal cord compression to bridge to definitive treatment with either kyphoplasty or radiation therapy [8]. The definitive treatment of spinal cord compression is local therapy with conventional external beam radiation therapy or decompression surgery, depending on the patient's functional status [8]. The first-line systemic treatment approved for advanced HCC is a tyrosine kinase inhibitor targeting vascular endothelial growth factor receptors such as sorafenib, sunitinib, and lenvatinib [9]. Immunotherapy in advanced HCC has shown promising results. A phase III trial compared a combination of atezolizumab plus bevacizumab versus sorafenib alone showed significant improvement in overall survival and progression-free survival [10]. Based on these trials, the FDA approved the combination of atezolizumab plus bevacizumab to treat advanced HCC [11]. HCC patients can present with symptoms of spinal cord compression, and HCC should be included as one of the differentials in these patients.

## Conclusions

Hepatocellular cancer is an aggressive tumor with a wide array of presentations, but upfront presentation as spinal cord compression is rare and a neuro-oncological emergency. The case presents a diagnostic dilemma and poses a challenge to the treatment approach depending on the patient's performance status and quality of life.
